# Heat Shock Proteins and Exercise in Skeletal Muscle Insulin Resistance: Protective Mechanisms and Therapeutic Potential

**DOI:** 10.3390/ijms27156587

**Published:** 2026-07-24

**Authors:** Mariam A. Othman, Joo Hyun Kim, Khaled Y. Kamal, John M. Lawler

**Affiliations:** 1 Department of Kinesiology & Sport Management, School of Education and Human Development, Texas A&M University, College Station, TX 77845, USA; mariam.othman@tamu.edu (M.A.O.); dhwngusdh@tamu.edu (J.H.K.); 2 Department of Kinesiology and Health, Iowa State University, Ames, IA 50011, USA; 3 Department of Nutrition, Texas A&M University, College Station, TX 77840, USA

**Keywords:** skeletal muscles, heat shock proteins, exercise, insulin resistance, molecular pathways

## Abstract

Heat shock proteins (HSPs) are a family of conserved molecular chaperons present in both prokaryotic and eukaryotic species, playing a crucial role in maintaining cellular proteostasis and enhancing stress resilience. HSPs have a multitude of roles in regulating cell signaling transduction, antioxidant defenses, apoptosis, and protein folding, thereby contributing to overall cellular homeostasis. Insulin resistance is characterized by elevated oxidative stress, dysregulated pro-inflammatory signaling, and impaired cellular stress response, ultimately leading to deficient glucose uptake in skeletal muscle. Many studies have illustrated the benefits of exercise in improving insulin resistance and reducing the risk of metabolic disorders, such as type 2 diabetes. Habitual exercise and lifestyle modifications have been shown to activate heat shock response, enhancing HSP70 expression and promoting cellular adaptations that protect against metabolic dysfunction. However, the link between HSPs, particularly HSP70, and skeletal muscle insulin resistance remains complex and not fully elucidated. In this review, we discuss the mechanistic pathways by which HSP70 modulates insulin resistance, mitochondrial function, and inflammatory responses in skeletal muscle. Additionally, we discuss the protective effects of exercise-induced HSP70 expression and its potential as a therapeutic target for improving insulin sensitivity and metabolic health.

## 1. Introduction

Insulin resistance (IR), characterized by impaired insulin function in tissues like skeletal muscle, adipocytes, and the liver, impacts more than 86 million U.S. adults aged 20 or older [1]. Glucose uptake is diminished by insulin resistance in metabolically active tissues, leading to compensatory hyperinsulinemia and increased pancreatic β-cell insulin production [2]. Despite the prevalence of insulin resistance, individuals with it mostly go undiagnosed, with the condition persisting for 10–12 years. This prolonged period of undiagnosed insulin resistance contributes to metabolic dysfunction, increasing the risk of obesity, type 2 diabetes (T2D), and associated complications such as cardiovascular disease, neuropathy, and nephropathy [3]. Factors contributing to the development of insulin resistance include a sedentary lifestyle, caloric excess, poor dietary habits, obesity, genetic predisposition, and acute or chronic illness. Furthermore, insulin resistance can be induced secondary to pharmacological treatments, including glucocorticoids, antiretroviral therapy, and beta blockers [4].

In skeletal muscle, IR hampers the translocation and availability of glucose receptors (e.g., GLUT4), which are essential for glucose uptake, glycogen synthesis, and anabolic signaling. Impaired GLUT4 translocation leads to hyperglycemia and compensatory hyperinsulinemia, further exacerbating metabolic dysregulation [5,6]. Skeletal muscle accounts for approximately 80% of insulin-stimulated glucose uptake at rest. During exercise, insulin-dependent and insulin-independent pathways increase the translocation of GLUT4 to the sarcolemma and increase glucose uptake [7]. Disruption of GLUT4 function impairs metabolic homeostasis [8]. While exercise promotes insulin-independent upregulation of GLUT4 and glucose uptake, insulin resistance disrupts these mechanisms, leading to increased circulating free fatty acids (FFAs) and inflammatory cytokines, further propagating metabolic dysfunction [5,6]. Moreover, insulin acts as a vasoactive hormone, regulating its delivery to muscle tissues by promoting vasodilation [9]. However, insulin resistance compromises this process, diminishing nitric oxide-mediated vasorelaxation and impairing glucose delivery to skeletal muscles [10].

Substantial evidence supports the notion that the metabolic benefits of habitual exercise are partially due to non-pharmacological heat stress. Hooper [11] first demonstrated that passive heat therapy (e.g., hot tub immersion) for three weeks led to modest improvements in fasting plasma glucose and glycosylated hemoglobin levels in patients with T2D. In addition, Gupte et al. [12] reported that heat treatment prevented peripheral insulin resistance in rodents fed a high-fat diet, linked to in vivo activation of heat shock proteins (HSPs). Significant attention to HSPs, particularly HSP70, has focused on the ability to modulate metabolic and inflammatory pathways implicated in insulin resistance [1]. Indeed, HSP70 is hypothesized to counteract insulin resistance by suppressing pro-inflammatory kinase cascades (e.g., JNK, IKK-β), preserving mitochondrial function, and improving glucose uptake [10].

Studies have shown a strong association between HSP70 expression and healthy metabolism status, emphasizing the critical role of HSPs in maintaining mitochondrial homeostasis, preserving insulin, and increasing glucose uptake [13]. HSP70 exists in both inducible (Hsp70) and non-inducible (Hsc70) forms, with the inducible form, Hsp70, being upregulated under stress conditions, while the non-inducible form, Hsc70, is constitutively expressed [14]. Importantly, an in vivo imbalance between intracellular HSP70 extracellular HSP70 (iHSP70/eHSP70) may sustain low-grade inflammation in metabolic disorders, including T2D [15,16]. For example, intracellular HSP70 exerts anti-inflammatory control by inhibiting the transcription factor nuclear factor-kappa B (NF-κB), a key proinflammatory transcription factor implicated in insulin resistance [17]. In addition, HSP70 induced through heat stress protects against insulin resistance, possibly by suppressing inducible nitric oxide synthase (iNOS) [18]. Habitual physical activity improved HSP70 levels in skeletal muscles and downregulated iNOS, providing an in vivo mechanism for reduced inflammation and improved glucose uptake and metabolic homeostasis [7,19].

Indeed, both heat shock and exercise-induced upregulation of HSPs could inhibit key pathogenic drivers contributing to insulin resistance, such as oxidative stress and stress-activated kinase (e.g., JNK) [1,12,20]. However, while progress has been made in elucidating these mechanisms, further research is needed to fully characterize the molecular pathways linking HSP70 to metabolic health. Therefore, this review critically examines the molecular mechanisms through which exercise-induced HSP70 mitigates skeletal muscle insulin resistance, with emphasis on redox regulation, mitochondrial function, and inflammation. By integrating recent insights from molecular biology, exercise physiology, and metabolic signaling, this review will highlight HSP70 as a therapeutic strategy for combating insulin resistance and reduce the risk of metabolic disease.

## 2. Heat Shock Proteins (HSPs)

In 1962, Ferruccio Ritossa, an Italian scientist, accidentally raised the incubation temperature of *Drosophila* larvae and observed enhanced transcription of unknown genes. He proposed these proteins as HSPs [21]. HSPs are a highly conserved protein family known for their function as molecular chaperones that assist in protein folding, prevent misfolding, and facilitate proteostasis [22]. In addition, HSPs activate antioxidant defenses and cellular stress response pathways, enhancing cell survival under metabolic stress [23]. Heat shock proteins are classified into different families based on their molecular weight, including HSP100s, HSP90s, HSP70s, HSP60s, HSP40s, and some smaller HSPs (15–40 kDa) [24].

HSP70 is more commonly used in animal studies, and HSP72 in human studies. This review will use the term HSP70 throughout to refer specifically to the stress-inducible member of the HSP70 family to maintain consistency. The HSP70 family comprises distinct gene products that differ in their expression patterns and physiological roles. *HSPA1A* and *HSPA1B* (commonly termed HSP70-1 or HSP72) are the main stress-inducible isoforms, present at minimal or undetectable levels under basal conditions but significantly upregulated in response to cellular stress [25,26]. Depending on the cell type, *HSPA6* represents an even more tightly regulated isoform and is induced under conditions of severe physiological stress or specific chemical insult [27].

HSPs are induced by physiological or environmental stressors such as heat, cold, starvation, hypoxia, pathogen invasion, malnutrition, chemical exposure, or UV radiation [28,29,30,31]. This induction supports cell survival under adverse conditions by stabilizing proteins, facilitating proteasome-mediated degradation of damaged or marked proteins, and inhibiting apoptosis at mitochondrial and cytosolic levels [32]. HSP70, for example, promotes cellular tolerance to inflammatory cytokines and enhances heat tolerance in fibroblasts in vitro [33,34].

The HSP70 family is particularly well-recognized for its role in cellular proteostasis, stress protection, and survival [35]. Stress factors such as oxidative stress, inflammation, changes in calcium levels, lactic acidosis, ischemia/reperfusion, and energy depletion can induce HSP70 expression [36,37]. HSP70 interacts with key signaling pathways, including Akt-mTOR activation and skeletal muscle mass protection or enhancement. Elevation of HSP70 also inhibits NF-κB and FOXO signaling, thus limiting muscle atrophy while promoting regeneration [38,39,40].

HSP70 has distinct intracellular and extracellular functions. Intracellularly, it modulates anti-inflammatory mechanisms, maintains protein homeostasis, and regulates nuclear, cytosolic, and mitochondrial functions [41,42]. It also plays a crucial role in mitigating endoplasmic reticulum (ER) stress by supporting the unfolded protein response (UPR), preventing proteotoxicity, and reducing inflammation [43,44]. Through its regulation of ER chaperone activity and suppression of stress-induced JNK activation, HSP70 helps counteract insulin resistance and muscle dysfunction in metabolic disorders [45].

Extracellularly, HSP70 performs immunomodulatory functions, acting as a danger-associated molecular pattern (DAMP) that signals through pattern recognition receptors, thereby mediating immune tolerance or pro-inflammatory responses depending on the cellular context [41]. Collectively, the HSP70 network serves as a molecular safeguard, linking stress adaptation to metabolic regulation and forming the mechanistic foundation for its therapeutic relevance in insulin resistance and exercise physiology.

## 3. HSP70 and Its Role in Muscle and Metabolic Health

While HSP70 exerts broad cytoprotective effects across multiple tissues, including hepatic, cardiac, and immune cells, this section focuses specifically on its roles in skeletal muscle, the primary site of insulin-stimulated glucose disposal and a tissue susceptible to metabolic and mechanic stress.

Skeletal muscle accounts for approximately 80% of postprandial glucose uptake, making it central to the pathophysiology of insulin resistance and T2D diabetes. HSPs are essential regulators of metabolic health in skeletal muscles, and their cytoprotective functions help mitigate insulin resistance and prevent T2D by modulating cellular stress responses in vivo [46,47]. Beyond its general stress-protective role, HSP70 plays a crucial role in muscle maintenance and regeneration. It supports muscle fiber size, protects against muscle atrophy during unloading, and promotes recovery following injury [38,39]. Notably, HSP70 also plays an essential role in maintaining muscle fiber size during rest and mitigating atrophy during disuse. Inactivity in adult and elderly rats reduces HSP70 levels, but restoring its expression significantly minimizes fiber atrophy [39].

At the level of intracellular signaling, HSP70 interacts with and stimulates the Akt-mTOR pathway in skeletal muscle, a critical anabolic regulator of skeletal muscle protein synthesis and maintenance. Specifically, HSP70 facilitates Akt phosphorylation, which activates mTORC1 and downstream effectors that drive ribosomal biogenesis and translational efficiency of contractile proteins [38]. Simultaneously, HSP70 suppresses two major atrophic signaling arms, NF-κB and FOXO signaling, preventing catabolic processes, reducing muscle atrophy, and enhancing muscle regeneration [39,40].

Recent studies reveal that HSP70 prevents insulin resistance and T2D through its anti-inflammatory, oxidative stress-reducing, and mitochondrial regulatory functions [46,47]. Specifically, HSP70 participates in the preservation and protection of metabolically active tissues, where it reduces inflammation [33], alleviates oxidative stress [13], enhances mitochondrial function [48], and maintains proteostasis [37]. These mechanisms are interconnected, and together they establish HSP70 as a molecular target linking cellular stress adaptation to whole-body metabolic regulation.

### 3.1. Anti-Inflammatory Properties

In skeletal muscle, chronic low-grade inflammation is recognized as a driver of insulin resistance, impaired satellite cell function, and progressive fiber atrophy, while HSP70’s anti-inflammatory properties are documented in multiple tissues; its role in resolving the intramuscular inflammatory signaling is specifically relevant to metabolic health.

HSPs exhibit anti-inflammatory effects by modulating macrophage polarization and promoting the folding of immune cell receptors, thereby triggering innate and adaptive immune responses. HSP70 enhances anti-inflammatory cytokine production in chronic inflammatory diseases [49]. The absence of HSP70 downregulates CD11b and CD68 mRNA expression in vitro early post-injury, impairing the regulation of inflammation and myoblast differentiation, which suggests its critical role in the inflammatory and repair processes [50,51].

Furthermore, HSPs regulate inflammation by modulating the pro-inflammatory c-Jun N-terminal kinase (JNK), which activates macrophages and promotes cytokine release. Increased JNK activation contributes to insulin resistance by impairing insulin signaling through phosphorylation of IRS-1 serine residues [52]. HSP70 induction suppresses JNK activation, improves insulin sensitivity, and reduces mitochondrial dysfunction and reactive oxygen species (ROS) production [1,53]. Pharmacological activation of HSP70 has shown promising metabolic outcomes, particularly in high-fat-diet-fed models [54].

### 3.2. HSP70 Regulation of Mitochondrial Function

Mitochondrial dysfunction is a hallmark of insulin resistance, marked by reduced ATP production and impaired β-oxidation [55]. HSP70 preserves mitochondrial integrity by facilitating the import of nuclear-encoded proteins and mitigating damage caused by oxidative stress [56]. Heat stress and HSP70 activation enhance mitochondrial enzyme activity, oxidative capacity, and insulin sensitivity, protecting against skeletal muscle insulin resistance [53,57]. Additionally, HSP70 also supports mitochondrial quality through mitophagy [58], a process of removing dysfunctional mitochondria. Reduced HSP70 expression limits this capacity, leading to mitochondrial dysfunction in insulin resistance [48]. Elevated HSP70 increases AMPK and SIRT1 activity, promoting mitochondrial biogenesis, fatty acid oxidation, and better insulin sensitivity [59].

The association between mitochondrial dysfunction and insulin resistance with HSP70 has spurred research into the role of HSP70 in enhancing mitochondrial function, especially during nutrient excess [12,37]. Studies show that heat therapy and exercise training (which produces significant heat) improve the mitochondrial biogenesis, mitochondrial enzyme activity, oxidative potential, and thus endurance capacity of muscle cells in vitro [60,61]. Elevated HSP70 levels correlate with a higher proportion of oxidative fiber types in skeletal muscles and enhanced mitochondrial enzyme activity [13].

Additionally, HSP70 overexpression has been shown to protect against high-fat-diet-induced impairments in skeletal muscle metabolism, promoting insulin sensitivity and glucose tolerance [62]. Exercise complements this process by facilitating glucose uptake in skeletal muscles through the translocation of GLUT4 to the cell membrane, a mechanism similar to insulin-stimulated glucose uptake and critical for individuals with reduced glucose utilization, such as those with T2D [63,64]. This highlights the multifaceted role of HSP70 in mitochondrial function, metabolic health, and glucose homeostasis.

## 4. Exercise-Induced Expression of Heat Shock Proteins (HSPs)

Exercise is a potent physiological stressor that induces the expression of HSPs, particularly HSP70, which acts as a universal stress (e.g., mechanical, metabolic, heat) indicator and plays a critical role in cellular homeostasis and adaptation to stress [62,65]. Elevated levels of HSP70 are observed following aerobic and resistance training, with its expression correlating positively with exercise intensity, duration, and training status [66,67]. Exercise-induced HSP70 expression facilitates cellular protection by stabilizing myofibrillar structures, supporting protein folding, and mitigating oxidative stress, thereby preserving muscle integrity [68,69]. Notably, HSP70 expression is influenced by training modality, fitness levels, and environmental conditions (e.g., heat stress), highlighting its adaptive role in metabolic and cellular resilience during exercise [1,70].

Both acute and chronic exercise paradigms, particularly endurance-type running, induce the expression of HSPs in various tissues, including skeletal muscle, liver, and myocardium [31]. For instance, subjects completed a 45-min running exercise at an intensity corresponding to 75% of their VO_2_max [71]. A single bout of treadmill running increases HSP70 mRNA and protein levels in murine skeletal muscle, cardiac muscle, as well as hepatic tissue [72]. The mechanisms triggering HSP production during exercise include increased tissue temperature, oxidative stress, depletion of glucose and glycogen storage, ischemia, hypoxia, altered calcium levels, and pH changes [73]. This stress-induced expression of HSPs, particularly HSP70, is crucial for cell protection, restoration of homeostasis, and adaptation to physiological stressors induced by exercise [74]. Exercise serves as a non-toxic stressor, providing insights into the regulation of HSP gene expression.

Exercise, especially strenuous aerobic activities like running, significantly impacts the levels of extracellular HSP70 in the blood [75]. Various studies have demonstrated that aerobic exercises can elevate cellular and blood eHSP-70 levels both during and post-exercise [76,77]. The rise in temperature and metabolism during exercise activates the heat shock response (HSR), leading to increased HSP levels. Interestingly, even human in vivo adipose tissue, which experiences lesser temperature increases during exercise, displays elevated and prolonged upregulation of HSP levels post-exercise, suggesting potential benefits against chronic inflammation associated with obesity [70].

HSP70 is believed to function in protecting cellular factors and sensing biochemical stressors. Highlighting its role as a universal stress indicator, exercise produces many cellular stressors, including heat, metabolism, energy, mechanical stress, and oxidative stress, thus providing a comprehensive and integrative perspective on stress [65]. Consequently, the combination of various stressors, such as physical exercise and changes in environmental conditions (e.g., heat, cold, altitude), further intensifies the upregulation of cellular HSP70 [78]. The body exerts increased physiological responses to manage and mitigate stress, particularly in the future. In prolonged or uncompensated stress, such as chronic inflammation, the ability to maintain homeostasis is compromised or overwhelmed, potentially leading to pathological conditions, including overtraining [79].

Experimental evidence from muscle and liver tissues demonstrates that oxidative load proportionally drives compensatory increase in HSP70 levels [71]. Functionally, HSP70 acts as an ATP-dependent chaperone, facilitating protein refolding and assisting in protein translocation across membranes, and is among the first responders to oxidative stress. They play a protective role by shielding enzymes and other proteins from the harmful effects of ROS [80].

The elevation of HSP70 with exercise is now recognized as a central mediator of the systemic metabolic benefits of physical training. Common challenges to tissues during exercise, such as mechanical stress, acidosis, hypoxia, ischemia, ROS formation, and changes in calcium signaling, are shown to induce HSP expression independent of the exercise [1]. Indeed, some of the HSP70 benefits of exercise arise from body temperature increases associated with prolonged or intense exercise. Human in vivo studies show that HSP70 induction via heat treatment or pharmacologic intervention, as well as transgenic overexpression in animals, results in metabolic enhancement [81].

### 4.1. Exercise Dose–Response Effects and Heat Shock Proteins

Exercise dose, defined by intensity, duration, and modality, critically determines the magnitude and temporal kinetics of HSP70 induction [1,75]. Adding complexity is the understanding that exercise-induced HSP expression depends on training modality, intensity, and duration [1]. For example, human in vivo aerobic exercises like running on a treadmill or downhill can increase cellular and blood HSP70 levels several times during and after exercise [76,77]. HSP70 and HSP90 concentrations also increase in skeletal muscle following a bout of exercise. For example, tissue hypoxia, characterized by lower-than-average oxygen content and pressure in the cell, may trigger HSP expression during exercise [82]. Consequently, duration and volume of exercise, exercise type and intensity, subjects’ training status, and environmental factors such as heat have been shown to affect the extent of HSP upregulation after exercise.

**Figure 1:**
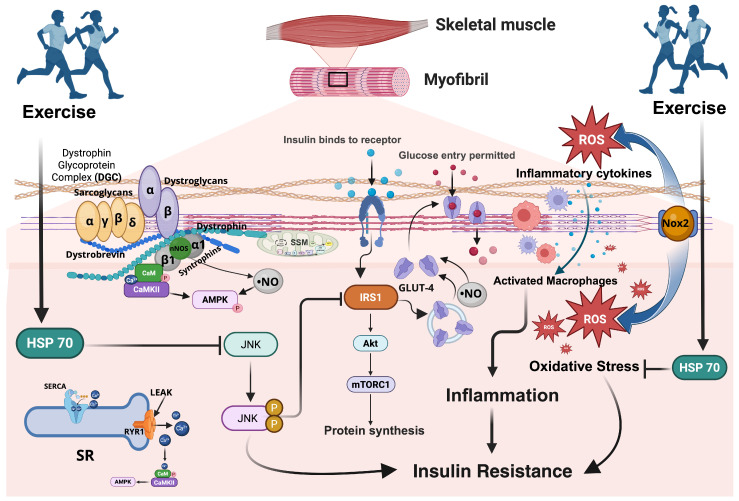
Integrated model depicting the molecular mechanisms through which exercise-induced HSP70 maintains skeletal muscle metabolic homeostasis. During exercise, multiple physiological stressors, including elevated temperature, calcium flux, and reactive oxygen species (ROS) production, activate signaling cascades that upregulate HSP70 expression in skeletal muscle. HSP70 acts as a cytoprotective molecular chaperone, stabilizing proteins, supporting mitochondrial integrity, and attenuating inflammatory signaling. Within the sarcolemma, activation of AMP-activated protein kinase (AMPK) and calcium/calmodulin-dependent kinase II (CaMKII) enhances nitric oxide (NO) bioavailability through neuronal nitric oxide synthase (nNOS), promoting glucose uptake via GLUT4 translocation. Concurrently, HSP70 inhibits JNK and nuclear factor-κB (NF-κB) pathways, which are otherwise activated by ROS and proinflammatory cytokines (e.g., TNF-α, IL-6) released from macrophages and damaged myofibers. This suppression of stress kinase activity preserves insulin receptor substrate-1 (IRS1) phosphorylation, sustaining downstream Akt–mTORC1 signaling and efficient glucose transport. By mitigating Nox2-derived oxidative stress, HSP70 prevents mitochondrial dysfunction and the propagation of chronic inflammation that drives insulin resistance. Collectively, the integration of mechanical, metabolic, and thermal cues during exercise elicits a coordinated HSP70-dependent adaptive response that counteracts oxidative stress and supports insulin sensitivity in skeletal muscle.

HSP70 expression in skeletal muscle is elevated following both aerobic and resistance training [83]. Crucially, HSP70 expression is influenced by exercise intensity. For example, there is a human in vivo positive correlation between HSP70 levels and exercise intensity during aerobic and resistance training [67,82]. This relationship is also observed when examining the connection between exercise intensity and metabolic outcomes, indicating that the induction of HSP70 may play a role in the metabolic benefits linked to exercise [66]. Most human studies of endurance aerobic and resistance anaerobic exercise focus on modulation of HSP70 in skeletal muscle and/or circulating monocytes [84]. HSP70 is the most abundant HSP and accounts for 1–2% of the cellular proteins commonly detected in skeletal muscle [16]. Aerobic exercise may enhance cellular survival, boost resistance to injury, and improve human adaptability in challenging environments [85]. The integrated molecular mechanisms underlying these exercise-induced adaptations are summarized in Figure 1, which depicts how HSP70 coordinates redox regulation, stress-kinase inhibition, and preservation of insulin signaling within skeletal muscle.

HSP70 expression also changes depending on the duration of the training regimen, distinguishing between acute and chronic training. Acute exercise sessions lead to significant increases in HSP70 levels within 24 h [86]. In contrast, chronic training regimens usually lead to only slight increases in HSP70 levels after each exercise bout [87]. Similarly, untrained individuals display lower baseline levels of HSP70 and show a more significant change in HSP expression after exercise compared to those who are fit [70]. However, when trained individuals stop exercising, their basal HSP expression levels will return to those similar to what was observed before exercise [1]. The inducing ability of iHSP70 has been demonstrated in various exercise protocols, including eccentric, concentric (non-damaging), aerobic, and resistance training, all of which can induce intramuscular HSP70 expression [88]. For instance, untrained subjects performed 50 high-force eccentric contractions with their non-dominant biceps brachii and ran downhill (−10°) for 30 min, significantly increasing HSP70. Muscle damage was indicated indirectly at 48 h post-exercise, as evidenced by loss of mobility, muscle soreness, and elevated serum creatine kinase activity in the biceps brachii [62]. Walsh et al. (2001) showed that moderate-intensity endurance exercise also significantly increases the concentration of circulating HSP70 in humans [77]. In rats, cardiac HSP70 was significantly elevated only when the animals were exercised (treadmill running) at 24 m/min and beyond. Rats ran on treadmills at 25 m/min and 0% grade until exhaustion. In the HIIT exhaustive exercise, rats ran on treadmills at 28 m/min and 0% grade for four periods of 10 min, interspersed with 10 min of rest, to approach 80% VO_2_max [86]. Although the increase in HSP70 was more significant in trained individuals compared to untrained individuals, aging is associated with a blunted increase in HSP70 after acute exercise in both untrained and trained elderly subjects [84].

Exercise-induced HSP70 responses are influenced by multiple variables, including exercise modality, intensity, duration, training status, tissue examined, and the timing of sample collection. To facilitate comparison among representative studies, Table 1 summarizes key experimental characteristics and the principal findings regarding exercise-induced HSP70 regulation.

#### 4.1.1. Exercise, HSP70, and GLUT4 Regulation: Relevance to Insulin Resistance

Over the past decade, the role of HSP70 in working muscle has become comprehensible: rather than being merely a general stress protein, it functions as a key regulator that stabilizes insulin signaling under physiological stress [37]. Elevated HSP70 can dampen stress kinases like JNK and IKK-β, usual suspects, when insulin signaling goes off track in obesity or during metabolic strain, thereby improving downstream insulin sensitivity [57]. That perspective aligns with reports positioning HSP70 as part of the cell’s metabolic housekeeping during training adaptations, not merely a damage signal [89].

Van Gerwen (2023) suggested that preserved Akt activity under stress is critical in enhancing GLUT4-dependent uptake, which relies on Akt-PI3K signaling [90]. In practical terms, HSP70 helps keep the early insulin nodes IRS-1 and the PI3K–Akt axis online, while HSP90 chaperones the LKB1-AMPK module that sustains exercise-responsive signaling; together these inputs converge on GLUT4 trafficking and membrane fusion [46,91,92].

**Table 1:** Representative evidence demonstrating exercise-induced HSP70 responses and their mechanistic relevance to skeletal muscle insulin sensitivity.

Experimental Model	Exercise Paradigm	Exercise Characteristics	Primary Tissue	HSP70 Response	Primary Mechanistic Effect	Physiological/Metabolic Outcome	Representative References
**Human**	Aerobic treadmill running	Acute, 45 min, ~75% VO_2_max	Skeletal muscle	↑ HSP70 mRNA and protein	Activation of the heat shock response, enhanced protein folding, maintenance of proteostasis, and improved cellular stress tolerance	Protection against acute exercise-induced metabolic and oxidative stress	[72]
**Human**	Endurance exercise	Moderate intensity, acute bout	Plasma/ circulation	↑ Extracellular HSP70	Activation of systemic heat shock signaling and intercellular stress communication	Enhanced systemic adaptation to physiological stress and improved metabolic resilience	[76,77,78]
**Human and** **Rodent**	Aerobic and resistance training	Moderate-to-vigorous intensity, repeated training	Skeletal muscle	Sustained elevation of basal HSP70	Improved protein quality control, reduced oxidative damage, attenuation of inflammatory signaling	Improved metabolic adaptation, enhanced exercise tolerance and greater insulin sensitivity	[66,67,68,69,70,83,84,85]
**Rodent**	Treadmill running	Acute endurance exercise	Skeletal muscle, heart, liver	↑ HSP70 expression	Heat- and exercise-mediated activation of HSF1 and molecular chaperone pathways	Protection against exercise-induced cellular stress and maintenance of tissue homeostasis	[71]
**Rodent**	Heat treatment ± exercise	Chronic intervention	Skeletal muscle	↑ HSP70	Suppression of JNK and IKKβ signaling, reduced inflammatory activation, preservation of insulin signaling	Prevention of diet-induced insulin resistance and improvement of glucose homeostasis	[1,12,53,54]
**Rodent**	Mechanical loading/ overload	Chronic Physiological loading	Skeletal muscle	↑ HSP70	Activation of Akt–mTOR signaling and inhibition of FOXO and NF-κB pathways	Preservation of muscle mass, reduced disuse atrophy, and enhanced muscle regeneration	[38,39,40]
**Human and** **Rodent**	Aerobic and resistance exercise	Acute and chronic training	Skeletal muscle	↑ HSP70	Preservation of mitochondrial integrity, enhanced oxidative enzyme activity, improved mitochondrial quality control and biogenesis	Increased oxidative capacity, improved metabolic flexibility, and enhanced mitochondrial function	[13,48,57,58,59,60,61]
**Human and** **Rodent**	Aerobic exercise	Acute and chronic exercise	Skeletal muscle	↑ HSP70	Stabilization of IRS-1/PI3K/Akt signaling, preservation of AMPK-mediated GLUT4 trafficking, reduction in ROS-induced impairment of insulin signaling	Increased GLUT4 translocation, enhanced glucose uptake, and improved insulin sensitivity	[48,63,92,93,94,95,96,97,98,99,100,101]

GLUT4 remains the central transporter of glucose into skeletal muscle, and is responsive to both insulin and contraction, and during exercise, it reaches the membrane through AMPK- and CaMKII-dependent routes that do not require insulin [7,92]. Because exercise activates AMPK, which can drive GLUT4 translocation on its own, HSP support of the LKB1–AMPK axis preserves that route and amplifies the glucose-uptake gain from training [7,48,92].

Training, of course, does more than move glucose; it reconditions the muscle’s stress responses. Part of that reconditioning is a quieter inflammatory backdrop: higher HSP70 often tracks with calmer NF-κB signaling and smoother insulin action, which in turn makes GLUT4 translocation less fragile [93]. This matters because contraction can spike ROS and activate NF-κB; when that spike is excessive or prolonged, insulin signaling and GLUT4 movement suffer, whereas HSP70/HSP72 can buffer the oxidative load and mute inflammatory cascades that otherwise derail glucose uptake [48,57,99].

On the energetics side, HSP70 lends a hand with protein quality control and mitochondrial upkeep, work that rarely grabs headlines but determines whether the cell can pay the ATP bill during repeated contractions [101]. HSP72 increases mitochondrial content and oxidative capacity and improves insulin sensitivity, which raises the ATP ceiling for motor-protein-driven vesicle transport and the SNARE-dependent fusion steps that complete GLUT4 delivery [48,57]. Hsc70 participates in the uncoating of clathrin-coated vesicles, feeding the recycling pool that supplies GLUT4, and small HSP-linked actin remodeling (via p38–HSP27) helps vesicles move along microtubules, dock, and finally fuse with the membrane [96]. These effects vary with exercise type, intensity, and duration, which together determine the acute molecular responses and long-term remodeling of muscle [92,97,102].

With longer programs, GLUT4 expression is elevated, and insulin sensitivity improves, benefits that are particularly visible in type 2 diabetes [100,103]. Mitochondria and capillaries usually follow suit, enhancing metabolic flexibility [98]. Together, HSP70 and GLUT4 exemplify the dual cellular protection and metabolic benefits of exercise [94,95].

#### 4.1.2. Insulin-Dependent Pathway During Exercise

When insulin levels decline during physical activity, the insulin signaling pathway continues to facilitate glucose uptake via its conventional cascade [95]. This involves activation of the insulin receptor and its downstream effector IRS-1, which stimulates PI3K, leading to the activation of Akt (protein kinase B) [104,105,106]. For Akt to become fully active, it must be phosphorylated at Ser473 by mTORC2, a step necessary for promoting GLUT4 translocation to the cell membrane and facilitating glucose transport into the cell [107,108]. Once activated, Akt phosphorylates AS160 (TBC1D4), a regulator of Rab GTPases, thereby reducing its inhibitory effect on GLUT4 vesicle trafficking and allowing those vesicles to merge with the plasma membrane [109,110].

Additionally, IGF-1 signaling also converges on the PI3K/Akt/mTOR pathway, enhancing both glucose uptake and promoting muscle growth and metabolic adaptations [111]. However, excessive mTORC1 activity, which can occur during nutrient excess, interferes with insulin sensitivity. This happens through serine phosphorylation of IRS-1, impairing its interaction with the insulin receptor and thereby disrupting signal transmission [112]. Moreover, Grb10, a protein stabilized by mTORC1, further impairs signaling by blocking the IR–IRS-1 interaction in vitro, exacerbating insulin resistance [113,114].

Conversely, mTORC2 serves a protective function within insulin signaling by enhancing Akt phosphorylation and maintaining glucose responsiveness in muscle and other tissues [108]. This interplay between mTORC1 and mTORC2 represents a critical regulatory balance that determines whether insulin signaling promotes efficient glucose utilization or leads to metabolic dysfunction [115].

#### 4.1.3. Insulin-Independent Pathway During Exercise

Exercise enhances glucose uptake through insulin-independent mechanisms, primarily involving AMPK, CaMKII, and nNOS, which respond to shifts in cellular calcium levels and energy status [116,117,118,119]. AMPK, activated during energy depletion (e.g., a high AMP/ATP ratio), facilitates GLUT4 translocation by phosphorylating targets like TBC1D1 and AS160 (TBC1D4), which regulate Rab GTPases responsible for vesicle movement to the cell membrane [114,120]. At the same time, CaMKII, which is activated by calcium influx during muscle contraction, contributes to GLUT4 mobilization by affecting the actin cytoskeleton and vesicle trafficking [121]. In addition, nNOS activity, stimulated by muscular workload, promotes glucose uptake through nitric oxide-mediated signaling, enhancing blood flow and nutrient delivery to active tissues [122].

These pathways also link to mitochondrial function through the activation of PGC-1α, a key regulator of mitochondrial biogenesis and oxidative metabolism, which is upregulated by both AMPK and CaMKII [123]. Furthermore, PGC-1α is controlled through two main mechanisms. One key level of regulation is transcriptional, where its gene expression is influenced by multiple transcription factors and external signals, such as insulin and glucagon levels, calcium concentrations, temperature changes, and physical activity, all acting through specific signaling pathways [124]. Collectively, these non-insulin-mediated routes ensure robust GLUT4 translocation and glucose uptake during periods of low insulin, such as prolonged or intense physical activity [5].

### 4.2. The Impact of Different Exercise Factors

Recent research suggests that aerobic capacity and exercise training significantly influence the expression of heat shock proteins (HSPs), particularly HSP70, which plays a key role in metabolic regulation [125]. Human or animal models with low aerobic capacity tend to show minute HSP induction, increasing their susceptibility to metabolic dysfunction [125]. For instance, rodent models bred for high or low running capacity demonstrate distinct metabolic responses, with high-capacity runners maintaining HSP70 expression and metabolic flexibility under dietary stress, whereas low-capacity runners require thermal interventions for similar protection [1,45,57].

Aerobic training significantly enhances GLUT4 expression and HSP70 levels, especially at moderate-to-high training volumes, while resistance training can also elevate these responses when performed at high volumes or in circuit-based protocols [100,126,127]. Chronic exercise has been shown to reduce HSP70 expression levels in subjects with metabolic dysfunction, as well as improve insulin sensitivity [1]. Both endurance and resistance training can induce HSP70, with eccentric running producing stronger responses than horizontal running [128]. Exercise intensity is a key factor; even a single bout of acute exercise increases HSP70 expression in an intensity-dependent method [84]. Higher-intensity endurance training elevates HSP70 more effectively than moderate activity [129]. Furthermore, high-intensity treadmill training significantly increases cardiac HSP70 compared to low-intensity voluntary exercise [130]. Moreover, extracellular HSP70 (eHSP70) release occurs during high-intensity exercise, further influencing immune regulation [131]. Moderate-to-high-intensity training over several weeks consistently increases HSP70 in leukocytes and skeletal muscle [89].

Duration also plays an important role, as acute exercise can increase HSP70 expression within a day [132], while long-term training elevates baseline HSP70 in muscle and cardiac tissue, but often blunts acute responses due to adaptation mechanisms such as the repeated bout effect [1]. Chronic training induces sustained GLUT4 expression and improves insulin sensitivity [48,100,133], whereas acute bouts only transiently increase GLUT4 translocation [7]. High-intensity interval training (HIIT) is particularly effective for enhancing GLUT4 responses, sometimes surpassing steady-state aerobic exercise [134]. Overall, these findings indicate that both intensity and duration of exercise, alongside genetic factors, are critical for modulating HSP70 expression and GLUT4 activity [131].

## 5. Conclusions

Insulin resistance has emerged as a major global health concern, significantly contributing to the increasing rates of metabolic diseases such as obesity and T2D. The development of insulin resistance is complex and multifactorial, with factors such as genetic predisposition, sedentary behavior, and excessive calorie intake playing key roles. In response to this challenge, research has identified exercise as a promising therapeutic approach. Exercise can effectively counteract insulin resistance by enhancing glucose uptake and improving insulin sensitivity.

Recent findings have highlighted the importance of heat shock proteins (HSPs), particularly HSP70, in mediating the positive effects of exercise on metabolic health. HSP70 contributes to cellular adaptation by offering protection against metabolic dysfunction. Specifically, HSP70 helps to reduce inflammation, oxidative stress, and mitochondrial dysfunction, all of which are critical contributors to the development of insulin resistance. HSP70 modulates a variety of cellular signaling pathways, including JNK, AMPK, and SIRT1. Through these pathways, HSP70 supports mitochondrial biogenesis and enhances metabolic efficiency. These mechanisms collectively protect against insulin resistance and glucose intolerance.

The degree to which exercise increases HSP70 expression is influenced by several factors, such as exercise intensity, modality, and duration. High-intensity exercise has been shown to produce the most significant increases in HSP70 levels in skeletal and cardiac muscle compared to moderate- or low-intensity exercise. Accordingly, gaining a deeper understanding of how exercise-induced modulation of HSP70 affects metabolic pathways will be instrumental in developing novel therapeutic approaches for preventing and treating insulin resistance, reducing the risk of metabolic disorders, and improving overall health outcomes.

Studies have found that HSP70 expression is generally higher in males, which suggests that men may benefit more from therapies that increase HSP70. However, further research is needed to understand how HSP70 functions in a sex-specific manner within skeletal muscle, with the goal of identifying the most effective therapeutic strategies for each sex. Additionally, future investigations should include both elderly and younger populations to account for their unique exercise protocols and physiological requirements.

## Data Availability

No new data were created or analyzed in this study. Data sharing is not applicable to this article.

## Abbreviations

The following abbreviations are used in this manuscript:

Akt	Protein kinase B; serine/threonine kinase central to insulin signaling and glucose uptake
AMPK	AMP-activated protein kinase; key energy sensor promoting glucose uptake and fatty-acid oxidation
AS160	Akt substrate of 160 kDa (also known as TBC1D4); regulates GLUT4 vesicle trafficking
ATP	Adenosine triphosphate; cellular energy currency
BGP-15	Hydroximic acid derivative and pharmacologic HSP co-inducer that improves insulin sensitivity
CaMKII	Calcium/calmodulin-dependent protein kinase II; mediates calcium signaling during muscle contraction
CK	Creatine kinase; marker of muscle damage
DAMPs	Damage-associated molecular patterns; endogenous molecules triggering immune activation
eHSP70	Extracellular heat shock protein 70; circulating form with immunomodulatory function
ER	Endoplasmic reticulum; site of protein folding and the unfolded-protein response (UPR)
FFA	Free fatty acid; lipid species contributing to insulin resistance when elevated
FOXO	Forkhead box O transcription factors; mediate muscle atrophy and oxidative stress responses
GLUT4	Glucose transporter type 4; insulin-responsive transporter in skeletal muscle and adipose tissue
HFD	High-fat diet; experimental model for inducing metabolic dysfunction
HSP	Heat shock protein; conserved molecular chaperone family aiding in protein folding and stress tolerance
HSP70 (Hsp70)	Inducible 70-kDa heat shock protein; central focus of this review
Hsc70	Heat shock cognate 70; constitutively expressed member of the HSP70 family
HSP72	Common designation for inducible HSP70 isoform in human studies
HSP90	90-kDa heat shock protein; stabilizes signaling proteins including kinases and steroid receptors
HSF1	Heat shock factor 1; transcriptional regulator of heat shock proteins
iHSP70	Inducible/intracellular heat shock protein 70; stress-responsive isoform
IGF-1	Insulin-like growth factor 1; anabolic growth factor activating PI3K/Akt/mTOR signaling
IL-6	Interleukin-6; cytokine modulating inflammation and metabolic signaling
iNOS	Inducible nitric oxide synthase; produces nitric oxide during inflammatory stress
IRS-1	Insulin receptor substrate-1; adaptor protein mediating insulin receptor signaling
JNK	c-Jun N-terminal kinase; stress-activated kinase impairing insulin signaling when overactive
LKB1	Liver kinase B1; upstream activator of AMPK
MAPK	Mitogen-activated protein kinase; family of kinases including ERK, JNK, and p38
mTOR	Mechanistic target of rapamycin; key regulator of growth and metabolism
mTORC1	mTOR complex 1; regulates protein synthesis and cell growth
mTORC2	mTOR complex 2; activates Akt by Ser473 phosphorylation
NF-κB	Nuclear factor-κB; transcription factor controlling inflammatory gene expression
nNOS	Neuronal nitric oxide synthase; enzyme generating NO in skeletal muscle
NO	Nitric oxide; vasodilator regulating blood flow and glucose delivery
Nox2	NADPH oxidase 2; enzyme complex generating reactive oxygen species (ROS)
PGC-1α	Peroxisome proliferator-activated receptor gamma coactivator-1 alpha; master regulator of mitochondrial biogenesis
PI3K	Phosphoinositide 3-kinase; mediates insulin signaling downstream of IRS-1
PKB	Protein kinase B (synonymous with Akt)
PPARα	Peroxisome proliferator-activated receptor alpha; nuclear receptor promoting fatty-acid oxidation
RNS	Reactive nitrogen species; nitrogen-derived oxidants affecting signaling and stress responses
ROS	Reactive oxygen species; oxygen-derived oxidants contributing to oxidative stress
SERCA	Sarco/endoplasmic reticulum Ca^2+^-ATPase; pumps calcium into SR for muscle relaxation
SIRT1	Sirtuin 1; NAD^+^-dependent deacetylase regulating mitochondrial function and metabolism
SR	Sarcoplasmic reticulum; intracellular Ca^2+^ storage organelle in muscle
T2D	Type 2 diabetes mellitus
TBC1D1	Tre-2/Bub2/Cdc16-domain family member 1; Rab GTPase-activating protein modulating GLUT4 vesicle traffic
TBC1D4	Tre-2/Bub2/Cdc16-domain family member 4 (also AS160); regulates GLUT4 translocation
TNF-α	Tumor necrosis factor-alpha; pro-inflammatory cytokine implicated in insulin resistance
UPR	Unfolded protein response; ER-stress pathway maintaining proteostasis
VO_2_max	Maximal oxygen consumption; measure of aerobic capacity

## References

[1] Archer A.E., Von Schulze A.T., Geiger P.C. (2018). Exercise, heat shock proteins and insulin resistance. Philos. Trans. R. Soc. Lond. Ser. B Biol. Sci..

[2] Freeman A.M., Pennings N. (2022). Insulin Resistance. StatPearls.

[3] Buscemi C., Randazzo C., Barile A.M., Bo S., Ponzo V., Caldarella R., Malavazos A.E., Caruso R., Colombrita P., Lombardo M. (2024). Factors associated with body weight gain and insulin-resistance: A longitudinal study. Nutr. Diabetes.

[4] Abdul-Ghani M.A., DeFronzo R.A. (2010). Pathogenesis of insulin resistance in skeletal muscle. J. Biomed. Biotechnol..

[5] Petersen M.C., Shulman G.I. (2018). Mechanisms of Insulin Action and Insulin Resistance. Physiol. Rev..

[6] Fujita S., Rasmussen B.B., Cadenas J.G., Grady J.J., Volpi E. (2006). Effect of insulin on human skeletal muscle protein synthesis is modulated by insulin-induced changes in muscle blood flow and amino acid availability. Am. J. Physiol. Endocrinol. Metab..

[7] Richter E.A. (2021). Is GLUT4 translocation the answer to exercise-stimulated muscle glucose uptake?. Am. J. Physiol. Endocrinol. Metab..

[8] Ormazabal V., Nair S., Elfeky O., Aguayo C., Salomon C., Zuñiga F.A. (2018). Association between insulin resistance and the development of cardiovascular disease. Cardiovasc. Diabetol..

[9] Manrique C., Sowers J.R. (2014). Insulin resistance and skeletal muscle vasculature: Significance, assessment and therapeutic modulators. Cardiorenal Med..

[10] Liu J., Liu Z. (2019). Muscle Insulin Resistance and the Inflamed Microvasculature: Fire from Within. Int. J. Mol. Sci..

[11] Hooper P.L. (1999). Hot-tub therapy for type 2 diabetes mellitus. N. Engl. J. Med..

[12] Gupte A.A., Bomhoff G.L., Swerdlow R.H., Geiger P.C. (2009). Heat treatment improves glucose tolerance and prevents skeletal muscle insulin resistance in rats fed a high-fat diet. Diabetes.

[13] Kuppuswami J., Senthilkumar G.P. (2023). Nutri-stress, mitochondrial dysfunction, and insulin resistance—Role of heat shock proteins. Cell Stress Chaperones.

[14] Drozdova P., Bedulina D., Madyarova E., Rivarola-Duarte L., Schreiber S., Stadler P.F., Luckenbach T., Timofeyev M. (2019). Description of strongly heat-inducible heat shock protein 70 transcripts from Baikal endemic amphipods. Sci. Rep..

[15] Lawler J.M., Kwak H.B., Song W., Parker J.L. (2006). Exercise training reverses downregulation of HSP70 and antioxidant enzymes in porcine skeletal muscle after chronic coronary artery occlusion. Am. J. Physiol. Regul. Integr. Comp. Physiol..

[16] Krause M., Heck T.G., Bittencourt A., Scomazzon S.P., Newsholme P., Curi R., de Bittencourt P.I.H. (2015). The Chaperone Balance Hypothesis: The Importance of the Extracellular to Intracellular HSP70 Ratio to Inflammation-Driven Type 2 Diabetes, the Effect of Exercise, and the Implications for Clinical Management. Mediat. Inflamm..

[17] Bittencourt A., Porto R.R. (2017). eHSP70/iHSP70 and divergent functions on the challenge: Effect of exercise and tissue specificity in response to stress. Clin. Physiol. Funct. Imaging.

[18] Morino S., Kondo T., Sasaki K., Adachi H., Suico M.A., Sekimoto E., Matsuda T., Shuto T., Araki E., Kai H. (2008). Mild electrical stimulation with heat shock ameliorates insulin resistance via enhanced insulin signaling. PLoS ONE.

[19] Song W., Kwak H.B., Kim J.H., Lawler J.M. (2009). Exercise training modulates the nitric oxide synthase profile in skeletal muscle from old rats. J. Gerontol. A Biol. Sci. Med. Sci..

[20] Lee W.J., Kim M., Park H.-S., Kim H.S., Jeon M.J., Oh K.S., Koh E.H., Won J.C., Kim M.-S., Oh G.T. (2006). AMPK activation increases fatty acid oxidation in skeletal muscle by activating PPARα and PGC-1. Biochem. Biophys. Res. Commun..

[21] Ritossa F. (1962). A new puffing pattern induced by temperature shock and DNP in *Drosophila*. Experientia.

[22] Xu Q., Metzler B., Jahangiri M., Mandal K. (2012). Molecular chaperones and heat shock proteins in atherosclerosis. Am. J. Physiol. Heart Circ. Physiol..

[23] Szyller J., Bil-Lula I. (2021). Heat Shock Proteins in Oxidative Stress and Ischemia/Reperfusion Injury and Benefits from Physical Exercises: A Review to the Current Knowledge. Oxidative Med. Cell Longev..

[24] Wang X., Chen M., Zhou J., Zhang X. (2014). HSP27, 70 and 90, anti-apoptotic proteins, in clinical cancer therapy. Int. J. Oncol..

[25] Rohde M., Daugaard M., Jensen M.H., Helin K., Nylandsted J., Jäättelä M. (2005). Members of the heat-shock protein 70 family promote cancer cell growth by distinct mechanisms. Genes Dev..

[26] Smith R.S., Meyers D.A., Peters S.P., Moore W.C., Wenzel S.A., Bleecker E.R., Hawkins G.A. (2007). Sequence analysis of HSPA1A and HSPA1B in a multi-ethnic study population. DNA Seq..

[27] Ramirez V.P., Stamatis M., Shmukler A., Aneskievich B.J. (2015). Basal and stress-inducible expression of HSPA6 in human keratinocytes is regulated by negative and positive promoter regions. Cell Stress Chaperones.

[28] Gbotsyo Y.A., Rowarth N.M., Weir L.K., MacRae T.H. (2020). Short-term cold stress and heat shock proteins in the crustacean Artemia franciscana. Cell Stress Chaperones.

[29] Nie H., Liu L., Wang H., Huo Z., Yan X. (2018). Stress levels over time in *Ruditapes philippinarum*: The effects of hypoxia and cold stress on hsp70 gene expression. Aquac. Rep..

[30] Roh J.S., Sohn D.H. (2018). Damage-associated molecular patterns in inflammatory diseases. Immune Netw..

[31] Jeyachandran S., Chellapandian H., Park K., Kwak I.S. (2023). A Review on the Involvement of Heat Shock Proteins (Extrinsic Chaperones) in Response to Stress Conditions in Aquatic Organisms. Antioxidants.

[32] Lanneau D., Brunet M., Frisan E., Solary E., Fontenay M., Garrido C. (2008). Heat shock proteins: Essential proteins for apoptosis regulation. J. Cell. Mol. Med..

[33] Li D.Y., Liang S., Wen J.H., Tang J.X., Deng S.L., Liu Y.X. (2022). Extracellular HSPs: The Potential Target for Human Disease Therapy. Molecules.

[34] Jagodzinski M., Hankemeier S., van Griensven M., Bosch U., Krettek C., Zeichen J. (2006). Influence of cyclic mechanical strain and heat of human tendon fibroblasts on HSP-72. Eur. J. Appl. Physiol..

[35] Morimoto R.I. (1991). Heat shock: The role of transient inducible responses in cell damage, transformation, and differentiation. Cancer Cells.

[36] Nollen E.A., Morimoto R.I. (2002). Chaperoning signaling pathways: Molecular chaperones as stress-sensingheat shock’proteins. J. Cell Sci..

[37] Hu C., Yang J., Qi Z., Wu H., Wang B., Zou F., Mei H., Liu J., Wang W., Liu Q. (2022). Heat shock proteins: Biological functions, pathological roles, and therapeutic opportunities. MedComm.

[38] Pomella S., Cassandri M., Antoniani F., Crotti S., Mediani L., Silvestri B., Medici M., Rota R., Rosa A., Carra S. (2023). Heat Shock Proteins: Important Helpers for the Development, Maintenance and Regeneration of Skeletal Muscles. Muscles.

[39] Dodd S., Hain B., Judge A. (2009). Hsp70 prevents disuse muscle atrophy in senescent rats. Biogerontology.

[40] Senf S.M., Dodd S.L., Judge A.R. (2010). FOXO signaling is required for disuse muscle atrophy and is directly regulated by Hsp70. Am. J. Physiol.-Cell Physiol..

[41] Mansilla M.J., Montalban X., Espejo C. (2012). Heat Shock Protein 70: Roles in Multiple Sclerosis. Mol. Med..

[42] Daugaard M., Rohde M., Jäättelä M. (2007). The heat shock protein 70 family: Highly homologous proteins with overlapping and distinct functions. FEBS Lett..

[43] Schroeder H.T., De Lemos Muller C.H., Heck T.G., Krause M., Homem de Bittencourt P.I. (2024). Heat shock response during the resolution of inflammation and its progressive suppression in chronic-degenerative inflammatory diseases. Cell Stress Chaperones.

[44] Chen D., Wang Y., Chin E.R. (2015). Activation of the endoplasmic reticulum stress response in skeletal muscle of G93A*SOD1 amyotrophic lateral sclerosis mice. Front. Cell Neurosci..

[45] Henstridge D.C., Whitham M., Febbraio M.A. (2014). Chaperoning to the metabolic party: The emerging therapeutic role of heat-shock proteins in obesity and type 2 diabetes. Mol. Metab..

[46] Chung J., Nguyen A.-K., Henstridge D.C., Holmes A.G., Chan M.H.S., Mesa J.L., Lancaster G.I., Southgate R.J., Bruce C.R., Duffy S.J. (2008). HSP72 protects against obesity-induced insulin resistance. Proc. Natl. Acad. Sci. USA.

[47] Rowles J.E., Keane K.N., Gomes Heck T., Cruzat V., Verdile G., Newsholme P. (2020). Are Heat Shock Proteins an Important Link between Type 2 Diabetes and Alzheimer Disease?. Int. J. Mol. Sci..

[48] Drew B.G., Ribas V., Le J.A., Henstridge D.C., Phun J., Zhou Z., Soleymani T., Daraei P., Sitz D., Vergnes L. (2014). HSP72 is a mitochondrial stress sensor critical for Parkin action, oxidative metabolism, and insulin sensitivity in skeletal muscle. Diabetes.

[49] Borges T.J., Wieten L., van Herwijnen M.J., Broere F., van der Zee R., Bonorino C., van Eden W. (2012). The anti-inflammatory mechanisms of Hsp70. Front. Immunol..

[50] Senf S.M., Howard T.M., Ahn B., Ferreira L.F., Judge A.R. (2013). Loss of the inducible Hsp70 delays the inflammatory response to skeletal muscle injury and severely impairs muscle regeneration. PLoS ONE.

[51] Fan W., Gao X.K., Rao X.S., Shi Y.P., Liu X.C., Wang F.Y., Liu Y.F., Cong X.X., He M.Y., Xu S.B. (2018). Hsp70 Interacts with Mitogen-Activated Protein Kinase (MAPK)-Activated Protein Kinase 2 To Regulate p38MAPK Stability and Myoblast Differentiation during Skeletal Muscle Regeneration. Mol. Cell. Biol..

[52] Mulyani W.R.W., Sanjiwani M.I.D., Sandra S., Prabawa I.P.Y., Lestari A.A.W., Wihandani D.M., Suastika K., Saraswati M.R., Bhargah A., Manuaba I. (2020). Chaperone-Based Therapeutic Target Innovation: Heat Shock Protein 70 (HSP70) for Type 2 Diabetes Mellitus. Diabetes Metab. Syndr. Obes..

[53] Geiger P.C., Gupte A.A. (2011). Heat shock proteins are important mediators of skeletal muscle insulin sensitivity. Exerc. Sport Sci. Rev..

[54] Adachi H., Kondo T., Ogawa R., Sasaki K., Morino-Koga S., Sakakida M., Kawashima J., Motoshima H., Furukawa N., Tsuruzoe K. (2010). An acylic polyisoprenoid derivative, geranylgeranylacetone protects against visceral adiposity and insulin resistance in high-fat-fed mice. Am. J. Physiol. Endocrinol. Metab..

[55] Takano C., Ogawa E., Hayakawa S. (2023). Insulin Resistance in Mitochondrial Diabetes. Biomolecules.

[56] Young J.C., Hoogenraad N.J., Hartl F.U. (2003). Molecular chaperones Hsp90 and Hsp70 deliver preproteins to the mitochondrial import receptor Tom70. Cell.

[57] Henstridge D.C., Bruce C.R., Drew B.G., Tory K., Kolonics A., Estevez E., Chung J., Watson N., Gardner T., Lee-Young R.S. (2014). Activating HSP72 in rodent skeletal muscle increases mitochondrial number and oxidative capacity and decreases insulin resistance. Diabetes.

[58] Song T., Yin F., Wang Z., Zhang H., Liu P., Guo Y., Tang Y., Zhang Z. (2023). Hsp70-Bim interaction facilitates mitophagy by recruiting parkin and TOMM20 into a complex. Cell. Mol. Biol. Lett..

[59] Boutant M., Cantó C. (2014). SIRT1 metabolic actions: Integrating recent advances from mouse models. Mol. Metab..

[60] Halling J.F., Jessen H., Nøhr-Meldgaard J., Buch B.T., Christensen N.M., Gudiksen A., Ringholm S., Neufer P.D., Prats C., Pilegaard H. (2019). PGC-1α regulates mitochondrial properties beyond biogenesis with aging and exercise training. Am. J. Physiol.-Endocrinol. Metab..

[61] Egawa T., Ohno Y., Goto A., Ikuta A., Suzuki M., Ohira T., Yokoyama S., Sugiura T., Ohira Y., Yoshioka T. (2014). AICAR-induced activation of AMPK negatively regulates myotube hypertrophy through the HSP72-mediated pathway in C2C12 skeletal muscle cells. Am. J. Physiol.-Endocrinol. Metab..

[62] Henstridge D.C., Febbraio M.A., Hargreaves M. (2016). Heat shock proteins and exercise adaptations. Our knowledge thus far and the road still ahead. J. Appl. Physiol..

[63] Alagar Boopathy L.R., Jacob-Tomas S., Alecki C., Vera M. (2022). Mechanisms tailoring the expression of heat shock proteins to proteostasis challenges. J. Biol. Chem..

[64] Gurley J.M., Griesel B.A., Olson A.L. (2016). Increased skeletal muscle GLUT4 expression in obese mice after voluntary wheel running exercise is posttranscriptional. Diabetes.

[65] Webb R., Hughes M.G., Thomas A.W., Morris K. (2017). The Ability of Exercise-Associated Oxidative Stress to Trigger Redox-Sensitive Signalling Responses. Antioxidants.

[66] Houmard J.A., Tanner C.J., Slentz C.A., Duscha B.D., McCartney J.S., Kraus W.E. (2004). Effect of the volume and intensity of exercise training on insulin sensitivity. J. Appl. Physiol..

[67] Liu Y., Lormes W., Wang L., Reissnecker S., Steinacker J.M. (2004). Different skeletal muscle HSP70 responses to high-intensity strength training and low-intensity endurance training. Eur. J. Appl. Physiol..

[68] Paulsen G., Vissing K., Kalhovde J.M., Ugelstad I., Bayer M.L., Kadi F., Schjerling P., Hallén J., Raastad T. (2007). Maximal eccentric exercise induces a rapid accumulation of small heat shock proteins on myofibrils and a delayed HSP70 response in humans. Am. J. Physiol. Regul. Integr. Comp. Physiol..

[69] Thompson H.S., Maynard E.B., Morales E.R., Scordilis S.P. (2003). Exercise-induced HSP27, HSP70 and MAPK responses in human skeletal muscle. Acta Physiol. Scand..

[70] Morton J.P., Maclaren D.P., Cable N.T., Campbell I.T., Evans L., Kayani A.C., McArdle A., Drust B. (2008). Trained men display increased basal heat shock protein content of skeletal muscle. Med. Sci. Sports Exerc..

[71] Salo D.C., Donovan C.M., Davies K.J. (1991). HSP70 and other possible heat shock or oxidative stress proteins are induced in skeletal muscle, heart, and liver during exercise. Free Radic. Biol. Med..

[72] Dozic S., Howden E.J., Bell J.R., Mellor K.M., Delbridge L.M.D., Weeks K.L. (2023). Cellular Mechanisms Mediating Exercise-Induced Protection against Cardiotoxic Anthracycline Cancer Therapy. Cells.

[73] Furrer R., Hawley J.A., Handschin C. (2023). The molecular athlete: Exercise physiology from mechanisms to medals. Physiol. Rev..

[74] Bonello J.P., Locke M. (2019). HSP72 expression is specific to skeletal muscle contraction type. Cell Stress Chaperones.

[75] Ogawa K., Seta R., Shimizu T., Shinkai S., Calderwood S.K., Nakazato K., Takahashi K. (2011). Plasma adenosine triphosphate and heat shock protein 72 concentrations after aerobic and eccentric exercise. Exerc. Immunol. Rev..

[76] Peake J., Nosaka K., Suzuki K. (2005). Characterization of inflammatory responses to eccentric exercise in humans. Exerc. Immunol. Rev..

[77] Walsh R.C., Koukoulas I., Garnham A., Moseley P.L., Hargreaves M., Febbraio M.A. (2001). Exercise increases serum Hsp72 in humans. Cell Stress Chaperones.

[78] Edwards J.P., Walsh N.P., Diment P.C., Roberts R. (2018). Anxiety and perceived psychological stress play an important role in the immune response after exercise. Exerc. Immunol. Rev..

[79] Krüger K., Reichel T., Zeilinger C. (2019). Role of heat shock proteins 70/90 in exercise physiology and exercise immunology and their diagnostic potential in sports. J. Appl. Physiol..

[80] Kurashova N.A., Madaeva I.M., Kolesnikova L.I. (2020). Expression of HSP70 Heat-Shock Proteins under Oxidative Stress. Adv. Gerontol..

[81] Henstridge D.C., Forbes J.M., Penfold S.A., Formosa M.F., Dougherty S., Gasser A., de Courten M.P., Cooper M.E., Kingwell B.A., de Courten B. (2010). The relationship between heat shock protein 72 expression in skeletal muscle and insulin sensitivity is dependent on adiposity. Metabolism.

[82] Liu Y., Steinacker J.M. (2001). Changes in skeletal muscle heat shock proteins: Pathological significance. Front. Biosci..

[83] Murlasits Z., Cutlip R.G., Geronilla K.B., Rao K.M., Wonderlin W.F., Alway S.E. (2006). Resistance training increases heat shock protein levels in skeletal muscle of young and old rats. Exp. Gerontol..

[84] Dimauro I., Mercatelli N., Caporossi D. (2016). Exercise-induced ROS in heat shock proteins response. Free Radic. Biol. Med..

[85] Yamada P., Amorim F., Moseley P., Schneider S. (2008). Heat Shock Protein 72 Response to Exercise in Humans. Sports Med..

[86] Milne K.J., Noble E.G. (2002). Exercise-induced elevation of HSP70 is intensity dependent. J. Appl. Physiol..

[87] Smolka M.B., Zoppi C.C., Alves A.A., Silveira L.R., Marangoni S., Pereira-Da-Silva L., Novello J.C., Macedo D.V. (2000). HSP72 as a complementary protection against oxidative stress induced by exercise in the soleus muscle of rats. Am. J. Physiol. Regul. Integr. Comp. Physiol..

[88] Milne K.J., Wolff S., Noble E.G. (2012). Myocardial accumulation and localization of the inducible 70-kDa heat shock protein, Hsp70, following exercise. J. Appl. Physiol..

[89] Costa-Beber L.C., Heck T.G., Fiorin P.B.G., Ludwig M.S. (2021). HSP70 as a biomarker of the thin threshold between benefit and injury due to physical exercise when exposed to air pollution. Cell Stress Chaperones.

[90] van Gerwen J., Shun-Shion A.S., Fazakerley D.J. (2023). Insulin signalling and GLUT4 trafficking in insulin resistance. Biochem. Soc. Trans..

[91] Kondo M., Kamiya H., Himeno T., Naruse K., Nakashima E., Watarai A., Shibata T., Tosaki T., Kato J., Okawa T. (2015). Therapeutic efficacy of bone marrow-derived mononuclear cells in diabetic polyneuropathy is impaired with aging or diabetes. J. Diabetes Investig..

[92] Richter E.A., Hargreaves M. (2013). Exercise, GLUT4, and skeletal muscle glucose uptake. Physiol. Rev..

[93] Angelini G., Castagneto-Gissey L., Salinari S., Bertuzzi A., Anello D., Pradhan M., Zschätzsch M., Ritter P., Le Roux C.W., Rubino F. (2022). Upper gut heat shock proteins HSP70 and GRP78 promote insulin resistance, hyperglycemia, and non-alcoholic steatohepatitis. Nat. Commun..

[94] Flores-Opazo M., McGee S.L., Hargreaves M. (2020). Exercise and GLUT4. Exerc. Sport Sci. Rev..

[95] Małkowska P. (2024). Positive Effects of Physical Activity on Insulin Signaling. Curr. Issues Mol. Biol..

[96] Morgan J.R., Prasad K., Jin S., Augustine G.J., Lafer E.M. (2001). Uncoating of clathrin-coated vesicles in presynaptic terminals: Roles for Hsc70 and auxilin. Neuron.

[97] Mukund K., Subramaniam S. (2020). Skeletal muscle: A review of molecular structure and function, in health and disease. Wiley Interdiscip. Rev. Syst. Biol. Med..

[98] Porter C., Reidy P.T., Bhattarai N., Sidossis L.S., Rasmussen B.B. (2015). Resistance Exercise Training Alters Mitochondrial Function in Human Skeletal Muscle. Med. Sci. Sports Exerc..

[99] Powers S.K., Jackson M.J. (2008). Exercise-induced oxidative stress: Cellular mechanisms and impact on muscle force production. Physiol. Rev..

[100] Sylow L., Kleinert M., Richter E.A., Jensen T.E. (2017). Exercise-stimulated glucose uptake-regulation and implications for glycaemic control. Nat. Rev. Endocrinol..

[101] Wang L., Schumann U., Liu Y., Prokopchuk O., Steinacker J.M. (2012). Heat shock protein 70 (Hsp70) inhibits oxidative phosphorylation and compensates ATP balance through enhanced glycolytic activity. J. Appl. Physiol..

[102] Lawler J.M., Rodriguez D.A., Hord J.M. (2016). Mitochondria in the middle: Exercise preconditioning protection of striated muscle. J. Physiol..

[103] Sticka K.D., Schnurr T.M., Jerome S.P., Dajles A., Reynolds A.J., Duffy L.K., Knall C.M., Dunlap K.L. (2018). Exercise Increases Glucose Transporter-4 Levels on Peripheral Blood Mononuclear Cells. Med. Sci. Sports Exerc..

[104] Zheng M., Wang P. (2021). Role of insulin receptor substance-1 modulating PI3K/Akt insulin signaling pathway in Alzheimer’s disease. 3 Biotech.

[105] Merz K.E., Thurmond D.C. (2020). Role of Skeletal Muscle in Insulin Resistance and Glucose Uptake. Compr. Physiol..

[106] Hers I., Tavaré J.M. (2005). Mechanism of feedback regulation of insulin receptor substrate-1 phosphorylation in primary adipocytes. Biochem. J..

[107] Chua B.T., Gallego-Ortega D., Ramirez de Molina A., Ullrich A., Lacal J.C., Downward J. (2009). Regulation of Akt(ser473) phosphorylation by choline kinase in breast carcinoma cells. Mol. Cancer.

[108] Albert V., Svensson K., Shimobayashi M., Colombi M., Muñoz S., Jimenez V., Handschin C., Bosch F., Hall M.N. (2016). mTORC2 sustains thermogenesis via Akt-induced glucose uptake and glycolysis in brown adipose tissue. EMBO Mol. Med..

[109] Sano H., Kane S., Sano E., Mîinea C.P., Asara J.M., Lane W.S., Garner C.W., Lienhard G.E. (2003). Insulin-stimulated Phosphorylation of a Rab GTPase-activating Protein Regulates GLUT4 Translocation. J. Biol. Chem..

[110] Eickelschulte S., Hartwig S., Leiser B., Lehr S., Joschko V., Chokkalingam M., Chadt A., Al-Hasani H. (2021). AKT/AMPK-mediated phosphorylation of TBC1D4 disrupts the interaction with insulin-regulated aminopeptidase. J. Biol. Chem..

[111] Chiu T.T., Sun Y., Koshkina A., Klip A. (2013). Rac-1 Superactivation Triggers Insulin-independent Glucose Transporter 4 (GLUT4) Translocation That Bypasses Signaling Defects Exerted by c-Jun N-terminal kinase (JNK)- and Ceramide-induced Insulin Resistance. J. Biol. Chem..

[112] Demir S., Nawroth P.P., Herzig S., Ekim Üstünel B. (2021). Emerging Targets in Type 2 Diabetes and Diabetic Complications. Adv. Sci..

[113] Yoneyama K., Mori N., Sato T., Yoda A., Xie X., Okamoto M., Iwanaga M., Ohnishi T., Nishiwaki H., Asami T. (2018). Conversion of carlactone to carlactonoic acid is a conserved function of MAX1 homologs in strigolactone biosynthesis. New Phytol..

[114] Edick A.M., Auclair O., Burgos S.A. (2020). Role of Grb10 in mTORC1-dependent regulation of insulin signaling and action in human skeletal muscle cells. Am. J. Physiol. Endocrinol. Metab..

[115] Szwed A., Kim E., Jacinto E. (2021). Regulation and metabolic functions of mTORC1 and mTORC2. Physiol. Rev..

[116] Hardie D.G. (2011). AMP-activated protein kinase: A cellular energy sensor with a key role in metabolic disorders and in cancer. Biochem. Soc. Trans..

[117] Habegger K.M., Hoffman N.J., Ridenour C.M., Brozinick J.T., Elmendorf J.S. (2012). AMPK enhances insulin-stimulated GLUT4 regulation via lowering membrane cholesterol. Endocrinology.

[118] Cartee G.D. (2015). Mechanisms for greater insulin-stimulated glucose uptake in normal and insulin-resistant skeletal muscle after acute exercise. Am. J. Physiol. Endocrinol. Metab..

[119] Araki S., Osuka K., Takata T., Tsuchiya Y., Watanabe Y. (2020). Coordination between Calcium/Calmodulin-Dependent Protein Kinase II and Neuronal Nitric Oxide Synthase in Neurons. Int. J. Mol. Sci..

[120] Treebak J.T., Glund S., Deshmukh A., Klein D.K., Long Y.C., Jensen T.E., Jørgensen S.B., Viollet B., Andersson L., Neumann D. (2006). AMPK-mediated AS160 phosphorylation in skeletal muscle is dependent on AMPK catalytic and regulatory subunits. Diabetes.

[121] Rose A.J., Richter E.A. (2005). Skeletal muscle glucose uptake during exercise: How is it regulated?. Physiology.

[122] Olver T.D., Laughlin M.H., Padilla J. (2019). Exercise and Vascular Insulin Sensitivity in the Skeletal Muscle and Brain. Exerc. Sport Sci. Rev..

[123] Qian L., Zhu Y., Deng C., Liang Z., Chen J., Chen Y., Wang X., Liu Y., Tian Y., Yang Y. (2024). Peroxisome proliferator-activated receptor gamma coactivator-1 (PGC-1) family in physiological and pathophysiological process and diseases. Signal Transduct. Target. Ther..

[124] Abu Shelbayeh O., Arroum T., Morris S., Busch K.B. (2023). PGC-1α Is a Master Regulator of Mitochondrial Lifecycle and ROS Stress Response. Antioxidants.

[125] Rogers R.S., Morris E.M., Wheatley J.L., Archer A.E., McCoin C.S., White K.S., Wilson D.R., Meers G.M., Koch L.G., Britton S.L. (2016). Deficiency in the Heat Stress Response Could Underlie Susceptibility to Metabolic Disease. Diabetes.

[126] Bird S.R., Hawley J.A. (2016). Update on the effects of physical activity on insulin sensitivity in humans. BMJ Open Sport Exerc. Med..

[127] Ren J.M., Semenkovich C.F., Gulve E.A., Gao J., Holloszy J.O. (1994). Exercise induces rapid increases in GLUT4 expression, glucose transport capacity, and insulin-stimulated glycogen storage in muscle. J. Biol. Chem..

[128] Lollo P.C., Moura C.S., Morato P.N., Amaya-Farfan J. (2013). Differential response of heat shock proteins to uphill and downhill exercise in heart, skeletal muscle, lung and kidney tissues. J. Sports Sci. Med..

[129] Milne S., Orbell S., Sheeran P. (2002). Combining motivational and volitional interventions to promote exercise participation: Protection motivation theory and implementation intentions. BR. J. Health Psychol..

[130] Heck T.G. (2023). What artificial intelligence knows about 70 kDa heat shock proteins, and how we will face this ChatGPT era. Cell Stress Chaperones.

[131] Heck T.G., Scomazzon S.P., Nunes P.R., Schöler C.M., da Silva G.S., Bittencourt A., Faccioni-Heuser M.C., Krause M., Bazotte R.B., Curi R. (2017). Acute exercise boosts cell proliferation and the heat shock response in lymphocytes: Correlation with cytokine production and extracellular-to-intracellular HSP70 ratio. Cell Stress Chaperones.

[132] Ogata T., Oishi Y., Higashida K., Higuchi M., Muraoka I. (2009). Prolonged exercise training induces long-term enhancement of HSP70 expression in rat plantaris muscle. Am. J. Physiol. Regul. Integr. Comp. Physiol..

[133] Konopka A.R., Douglass M.D., Kaminsky L.A., Jemiolo B., Trappe T.A., Trappe S., Harber M.P. (2010). Molecular adaptations to aerobic exercise training in skeletal muscle of older women. J. Gerontol. A Biol. Sci. Med. Sci..

[134] Gibala M.J., Little J.P., Macdonald M.J., Hawley J.A. (2012). Physiological adaptations to low-volume, high-intensity interval training in health and disease. J. Physiol..

